# The essential biology of the endoplasmic reticulum stress response for structural and computational biologists

**DOI:** 10.5936/csbj.201303010

**Published:** 2013-09-03

**Authors:** Sadao Wakabayashi, Hiderou Yoshida

**Affiliations:** a Department of Molecular Biochemistry, Graduate School of Life Science, University of Hyogo, Hyogo 678-1297, Japan

**Keywords:** ER stress, unfolded protein response, ATF6, IRE1, PERK, XBP1, ERAD

## Abstract

The endoplasmic reticulum (ER) stress response is a cytoprotective mechanism that maintains homeostasis of the ER by upregulating the capacity of the ER in accordance with cellular demands. If the ER stress response cannot function correctly, because of reasons such as aging, genetic mutation or environmental stress, unfolded proteins accumulate in the ER and cause ER stress-induced apoptosis, resulting in the onset of folding diseases, including Alzheimer's disease and diabetes mellitus. Although the mechanism of the ER stress response has been analyzed extensively by biochemists, cell biologists and molecular biologists, many aspects remain to be elucidated. For example, it is unclear how sensor molecules detect ER stress, or how cells choose the two opposite cell fates (survival or apoptosis) during the ER stress response. To resolve these critical issues, structural and computational approaches will be indispensable, although the mechanism of the ER stress response is complicated and difficult to understand holistically at a glance. Here, we provide a concise introduction to the mammalian ER stress response for structural and computational biologists.

## Introduction

**Figure 1:**
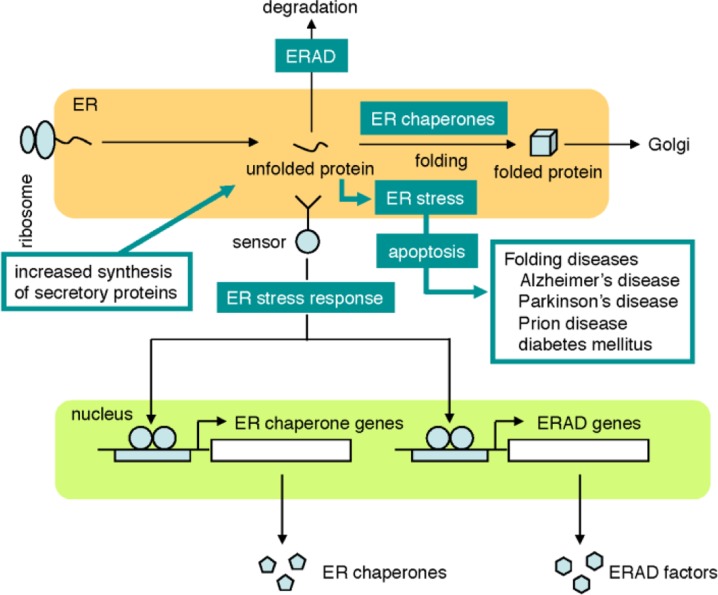
**The ER stress response.** The ER stress response is a cytoprotective mechanism that regulates the expression of ER chaperones as well as ERAD factors at the transcriptional level in accordance with cellular demands. When unfolded proteins accumulate in the ER, sensor molecules activate specific transcription factors, leading to transcriptional activation of genes encoding ER chaperones and ERAD factors. The ER stress response is thought to wane with aging, resulting in ER stress-induced apoptosis of cells and finally various diseases, which are collectively called “folding diseases”.

The endoplasmic reticulum (ER) is an organelle where secretory proteins are synthesized and folded with the assistance of ER chaperones, including BiP and calreticulin [1] (Figure 1). Correctly folded secretory proteins are transported to the Golgi apparatus, receive various modifications and are then secreted. Secretory proteins that cannot be properly folded in the ER are retrotranslocated to the cytoplasm by a mechanism called ER-associated degradation (ERAD) and degraded by the proteasome [2]. Thus, the function of ER chaperones and the ERAD system is important for homeostasis of the ER. When the synthesis of secretory proteins increases and overwhelms the capacity of ER chaperones and the ERAD, unfolded proteins accumulate in the ER and form aggregates (ER stress), which are highly toxic to cells and induce apoptosis [3]. Neurons are especially sensitive to ER stress, and ER stress can cause various neurodegenerative diseases, including Alzheimer's disease [4], Parkinson's disease [5] and prion disease [6]. ER stress also involved in the onset of other diseases such as diabetes mellitus [7–9], atherosclerosis [10], and UVA-induced cell damage [11]. These diseases caused by unfolded proteins are collectively called folding diseases or comformational diseases [12, 13].

To cope with ER stress, mammalian cells activate a cytoprotective mechanism called the ER stress response (also called the unfolded protein response) [14–18]. Upon activation of the ER stress response, the transcription of genes encoding ER chaperones and ERAD components is upregulated, resulting in increased expression of ER chaperones and ERAD components. It has been suggested that the ER stress response is attenuated with aging, and this is one of the diagnosis and treatment of folding diseases. Moreover, mammalian cells in which the ER capacity is artificially upregulated by manipulating the ER stress response could be useful for the production of large amounts of secretory proteins, including epidermal growth factor (EGF) and erythropoietin (EPO) in industry. Clarification of the mechanisms regulating the ER stress response is crucial to the above objectives, although there are numerous issues to be clarified; for example, the mechanism how molecular sensors detect ER stress (accumulation of unfolded proteins in the ER). In addition, acute ER stress induces the expression of ER chaperones and ERAD components for the survival of cells, whereas prolonged ER stress causes apoptosis to eliminate cells damaged by ER stress in order to ensure survival of the organism, but the mechanism that determines the cell fate (survival or cell death) has not been elucidated. Structural and computational approaches will be critical to decipher these critical problems.

In the following sections, we first describe the basic mechanism of the mammalian ER stress response and then briefly summarize the current status of structural and computational studies of the ER stress response. Because we have focused on the core story of the mammalian ER stress response, readers should also refer to the excellent review articles published recently for more detailed information on specific parts of the ER stress response.

## The basic mechanism of the mammalian ER stress response

The mammalian ER stress response consists of three pathways: the ATF6, IRE1 and PERK pathways, of which the main functions are augmentation of folding and ERAD capacity, and translational attenuation, respectively. Although these response pathways cross-talk with each other and have several branched subpathways, we focus on the main pathways in this section.

**Figure 2:**
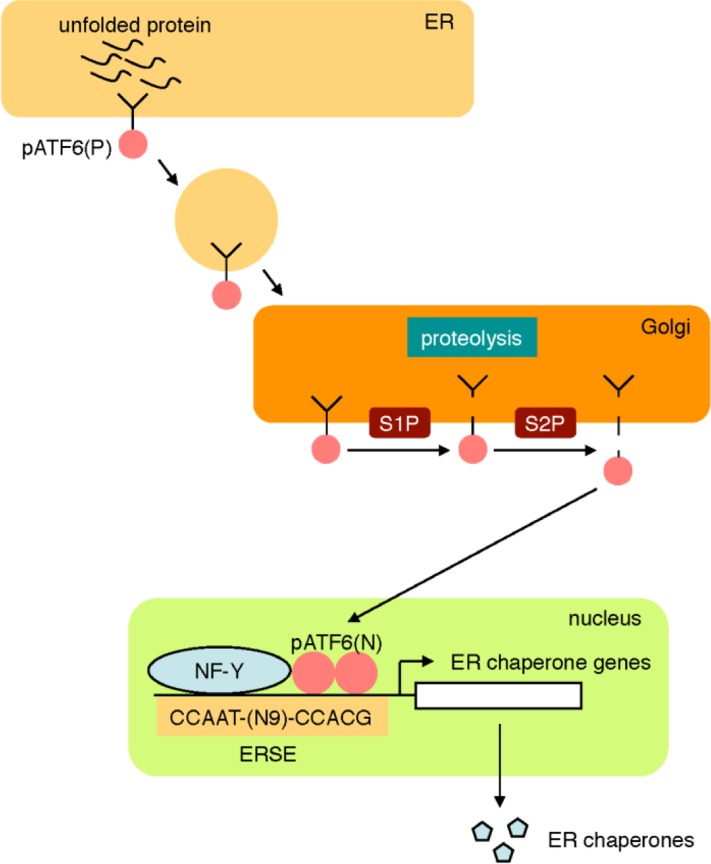
**The ATF6 pathway.** The sensor molecule pATF6(P) located on the ER membrane is transported to the Golgi apparatus by transport vesicles in response to ER stress. In the Golgi apparatus, pATF6(P) is sequentially cleaved by two proteases, S1P and S2P, resulting in release of the cytoplasmic portion pATF6(N) from the ER membrane. pATF6(N) translocates into the nucleus and activates transcription of ER chaperone genes through binding to the cis-acting enhancer ERSE.

The ATF6 pathway regulates the transcriptional induction of ER chaperone genes [19–23]. pATF6(P) is a sensor molecule comprising a type II transmembrane protein residing on the ER membrane (Figure 2). When pATF6(P) detects ER stress, the protein is transported to the Golgi apparatus through vesicular transport in a COP-II vesicle and sequentially cleaved by two proteases residing in the Golgi, namely site 1 protease (S1P) and site 2 protease (S2P) [24]. The cytoplasmic portion of pATF6(P) (pATF6(N)) is released from the Golgi membrane, translocates into the nucleus, binds to an enhancer element called the ER stress response element (ERSE), and activates the transcription of ER chaperone genes, including BiP, GRP94, calreticulin and protein disulfide isomerase (PDI) [19]. The consensus nucleotide sequence of ERSE is CCAAT(N9)CCACG, and pATF6(N) recognizes both the CCACG portion and another transcription factor NF-Y, which binds to the CCAAT portion [25]. NF-Y is a general transcription factor required for the transcription of various human genes [26].

Mammalian cells have two ATF6 genes, ATF6α and ATF6β[27]. Recently, it was reported that the double knockout of murine ATF6α and ATF6β resulted in embryonic lethality [22, 28, 29], and a similar knockout in medaka fish (*Oryzias latipes*) caused impaired notochord development [30, 31], indicating that the ATF6 pathway is essential for vertebrate embryogenesis. ATF6α and ATF6β are expressed ubiquitously, and mammalian cells have several genes similar to ATF6, of which the expression is restricted to specific tissues. OASIS and BBF2H7 contribute to the development of bone and cartilage, respectively [32–34], whereas CREB-H is specifically expressed in the liver and involved in inflammation [29]. Luman regulates the transcription of ERAD genes such as *Herp* and *EDEM* [35–37], whereas Tisp40 expression is restricted to the testis [38–40]. These tissue-specific ATF6 family proteins may be specialized for tissue-specific ER stress responses.

**Figure 3:**
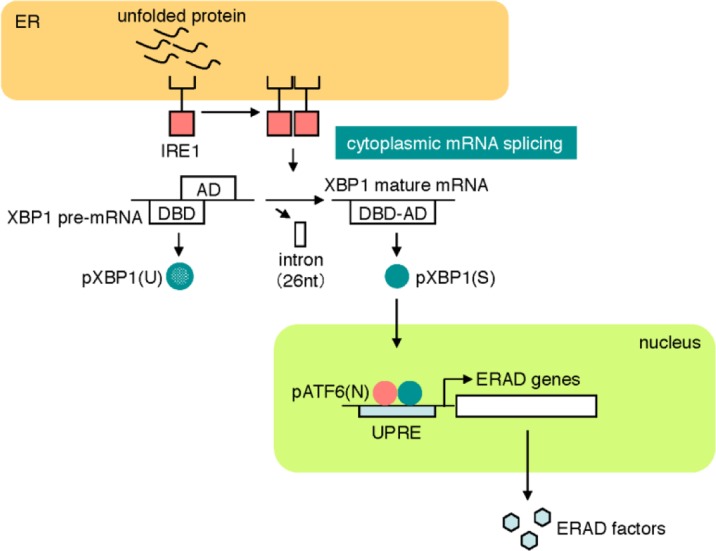
**The IRE1 pathway.** In normal growth conditions, the sensor molecule IRE1 is an inactive monomer, whereas IRE1 forms an active oligomer in response to ER stress. Activated IRE1 converts unspliced *XBP1* mRNA to mature mRNA by the cytoplasmic mRNA splicing. From mature *XBP1* mRNA, an active transcription factor pXBP1(S) is translated and activates the transcription of ERAD genes through binding to the enhancer UPRE.

The second pathway is the IRE1 pathway, which regulates the transcriptional induction of genes encoding ERAD components. IRE1 is a type I transmembrane protein residing on the ER membrane [41–44], of which the cytosolic portion contains kinase and RNase domains (Figure 3) [45–47]. IRE1 is an inactive monomer in normal growth conditions, whereas IRE1 becomes an active oligomer and forms clusters on the ER membrane in response to ER stress [48, 49]. IRE1 oligomers autophosphorylate each other to activate the RNase domain. IRE1 cleaves the pre-mRNA of *XBP*1 at two sites, and an unidentified RNA ligase ligates the two exons of the *XBP1* mRNA, resulting in splicing of *XBP1* mRNA and the excision of a small intron [50–52]. Because the length of the intron is 26 nt, splicing of *XBP1* mRNA by IRE1 causes a frame shift. Thus, the pre-mRNA and mature mRNA of *XBP1* encode different proteins, pXBP1(U) and pXBP1(S), respectively. pXBP1(S) is an active transcription factor and contains both of the DNA-binding domain and the transcriptional activation domain. pXBP1(S) forms a heterodimer with pATF6(N) and binds to the enhancer element called the unfolded protein response element (UPRE), resulting in the transcriptional activation of ERAD genes such as *HRD1*, *EDEM* and Derlins [53, 54]. pXBP1(S) is a very unstable protein degraded by the proteasome, and UBC9 protects it from degradation through direct binding [55].

Interestingly, the IRE1 pathway has unique features. Activated IRE1 degrades mRNAs associated with the ER membrane, which encode secretory proteins, in order to prevent further accumulation of unfolded proteins in the ER. This mechanism is called the regulated IRE1-dependent mRNA decay (RIDD) [49, 56]. In fission yeast *Schizosaccaromyces pombe*, RIDD is the main response mechanism of the ER stress response [57]. Moreover, the IRE1 pathway induces the apoptotic cascade through the TRAF2-ASK1-JNK pathway (see below). In addition, mammalian cells have two IRE1 genes, IRE1α and IRE1β. Both are involved in XBP1 splicing and RIDD, although IRE1β seems to be more closely involved with RIDD [58]. Expression of IRE1α is ubiquitous, whereas IRE1β is selectively expressed in the digestive tract and one of its main substrates is *mucin2* mRNA in Goblet cells [59]. The splicing of XBP1 mRNA by IRE1 is highly unusual, in that conventional mRNA splicing occurs in the nucleus and is catalyzed by the spliceosome, whereas splicing of *XBP1* mRNA takes place in the cytoplasm and the spliceosome is not involved [60]. The mechanism of cytoplasmic splicing is conserved from the yeast to mammals [61].

**Figure 4:**
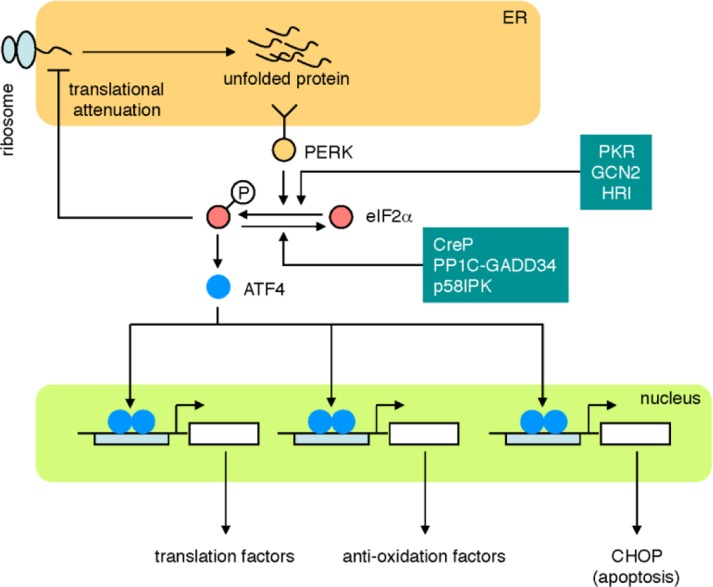
**The PERK pathway.** When PERK detects unfolded proteins in the ER, PERK phosphorylates eIF2α, resulting in translational attenuation and translational induction of ATF4. ATF4 activates the transcription of target genes encoding translation factors, anti-oxidation factors and a transcription factor CHOP. Other kinases such as PKR, GCN2 and HRI also phosphorylate eIF2α, and phosphorylated eIF2α is dephosphorylated by CReP, PP1C-GADD34 and p58IPK.

The third pathway is the PERK pathway (Figure 4). PERK is a sensor molecule residing on the ER membrane [62, 63]. The molecular structure of PERK is similar to that of IRE1, but the cytosolic domain of the PERK contains only the kinase domain. In the absence of ER stress, PERK is an inactive monomer, whereas PERK becomes an active oligomer upon ER stress, like IRE1. Activated PERK phosphorylates the α subunit of eukaryotic transcriptional initiation factor (eIF2α), resulting in the inactivation of eIF2α and translational attenuation, which prevents further accumulation of unfolded proteins in the ER. Interestingly, attenuation of general translation results in translational upregulation of ATF4 [64]. ATF4 is a transcription factor that binds to an enhancer element called the amino acid response element (AARE) and activates transcription of genes involved in translation [65] and anti-oxidative stress [66]. ATF4 also activates the apoptosis cascade by upregulating transcription of CHOP, a transcription factor involved in apoptosis. Phosphorylated eIF2α is gradually dephosphorylated by specific phosphatases such as CReP [67], PP1C-GADD34 [68] and p58IPK [69]. CReP is constitutively expressed, whereas transcription of GADD34 and p58IPK is upregulated by the PERK pathway and the ATF6 and IRE1 pathways, respectively.

Interestingly, eIF2α is also phosphorylated by other kinases, such as PKR, GCN2 and HRI [70]. Upon viral infection, PKR is activated and prevents production of viral proteins through eIF2α-mediated translational attenuation. When cellular amino acid levels drop, GCN2 is activated and halts translation by phosphorylating eIF2α. Hemoglobin consists of heme and globin, and when production of globin exceeds that of heme, HRI is activated and suppresses globin synthesis in erythrocytes by phosphorylating eIF2α. These subpathways of eIF2α-mediated translational attenuation are collectively called the integrated stress response [66].

Why does the mammalian ER stress response have multiple response pathways, namely the ATF6, IRE1 and PERK pathways? The answer has not been clarified, and we speculated that these multiple pathways functions consecutively in order to flexibly deal with ER stress [53]. The PERK pathway is a very rapid response pathway. Thus, the first response of mammalian cells is to attenuate translation and to refold unfolded proteins with already existing ER chaperones. If unfolded proteins still persist, cells activate the next pathway, the ATF6 pathway. Upon activation of this pathway, the folding capacity of the ER is augmented and many unfolded proteins would be refolded. The ATF6 pathway is relatively rapid but not so robust because pATF6(P) is consumed after cleavage by S1P and S2P. Thus, in the case that ER stress still persists, the IRE1 pathway is activated. The IRE1 pathway is not rapid but robust because pXBP1(S) activates its own transcription, leading to self amplification of the IRE1 pathway. If ER stress still has not been resolved, the apoptotic cascades are activated and cells damaged by ER stress are disposed [71, 72].

## The stress sensing mechanism of IRE1 and PERK

**Figure 5:**
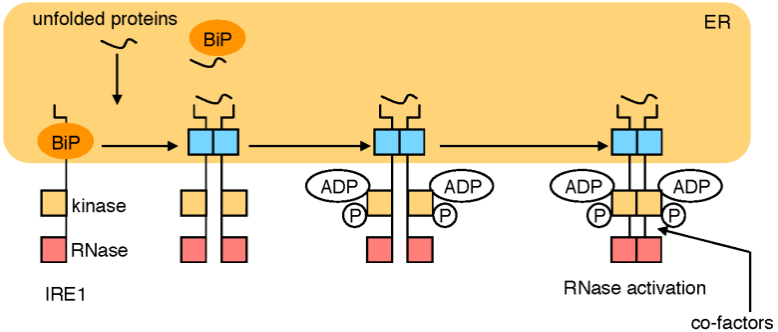
**Working hypothesis of the activation mechanism of IRE1.** In normal growth conditions, BiP binds to an IRE1 monomer and prevents IRE1 from forming an oligomer. Upon ER stress, BiP binds to unfolded proteins, and IRE1 forms an oligomer, to which unfolded proteins directly bind and cause a conformational change in IRE1. Then, ADP binds to IRE1 and IRE1 is transphosphorylated, resulting in activation of its RNase. Hypothetical cytosolic co-factors may bind to the cytoplasmic ligand-binding pocket and may modulate IRE1 activity.

The activation mechanisms of IRE1 and PERK are similar because the amino acid sequences of their luminal domains are similar. The current working hypothesis of the activation mechanism of IRE1 is as follows (Figure 5). In normal growth conditions, IRE1 is a monomer because BiP binds to the luminal domain of IRE1 and prevents it from forming an oligomer [48, 71–77]. Upon ER stress, BiP is sequestered to unfolded proteins accumulating in the ER lumen, and IRE1 is released from BiP. Then IRE1 forms a dimer, which binds unfolded proteins directly through a domain that is structurally similar to the antigen-peptide binding domain of the major histocompatibility complex (MHC) class-1 molecule [78]. This leads to oligomerization and clustering of IRE1 [48, 49, 79] and activation of the kinase domain of IRE1, which transphosphorylates IRE1. ADP-binding and transphosphorylation of IRE1 result in activation of the RNase domain, which cleaves the *XBP1(U)* mRNA and RIDD substrates.

Walter and colleagues analyzed the structure of the conserved core region of the Ire1p luminal domain (cLD) from the budding yeast *Saccharomyces cerevisiae* by X-ray crystallography, and showed that dimerization of cLD monomers creates a groove formed by α-helices and a β-sheet floor, which is reminiscent of the peptide-binding domains of the major histocompatibility complex (MHC) class-1 molecule [80]. They speculated that the binding of unfolded proteins to cLD changes the quaternary structure of IRE1, resulting in autophosphorylation of IRE1. On the contrary, Kaufman and colleagues determined the X-ray crystal structure of the luminal domain of human IRE1α and reported that dimerization of the luminal domains creates an MHC-like groove but this groove is too narrow for peptide binding, although it would be possible that the luminal domain changes its structure to accommodate unfolded proteins like the induced-fit model. Structural analysis of IRE1 bound to an unfolded protein would be helpful to resolve this important issue. Walter and colleagues also analyzed the crystal structure of the oligomer of the cytosolic domains of yeast Ire1p and found that they assemble like a rod, which may be important for autophosphorylation and the activation of RNase of yeast Ire1p [49]. Ron and colleagues analyzed the structure of a cocrystal of yeast Ire1p complexed with ADP and quercetin and revealed that the flavonol quercetin binds to the ligand-binding pocket at the dimer interface of the kinase extension nuclease domain of Ire1p, suggesting the existence of endogenous cytoplasmic ligands that may modulate Ire1p activity [81]. Sicheri and colleagues and Pearl and colleagues also reported the crystal structure of the cytoplasmic domain of yeast and human Ire1p, respectively [82, 83].

## The stress sensing mechanism of ATF6

**Figure 6:**
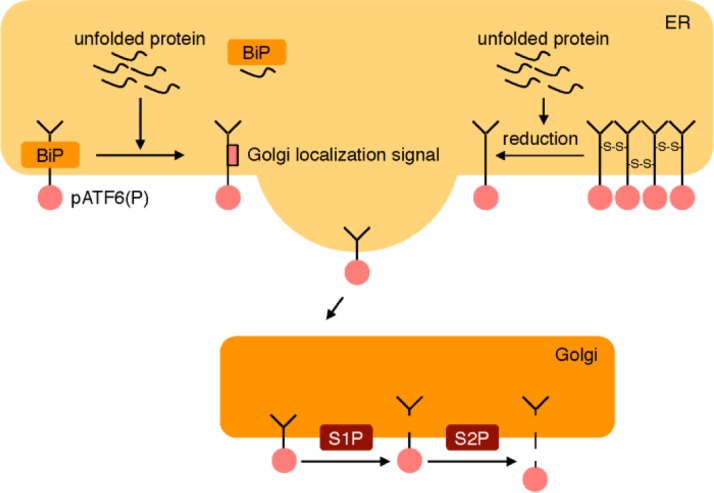
**Two working models of the activation mechanism of ATF6.** According to the first model, BiP binds to pATF6(P) and masks the Golgi localization signal in normal growth conditions. Upon ER stress, unfolded proteins sequester BiP from pATF6(P), resulting in translocation of pATF6(P) to the Golgi apparatus. In the second model, pATF6(P) forms an oligomer through disulfide bonds and is anchored in the ER membrane, while disulfide bonds are cleaved by reduction upon ER stress and a pATF6(P) monomer translocates to the Golgi apparatus. Both models are not mutually exclusive.

The sensor molecule of the ATF6 pathway is pATF6(P), which is a type II transmembrane protein with one transmembrane domain. Prywes and colleagues reported that BiP binds to the luminal domain of pATF6(P), which masks the Golgi-localization signal and keeps pATF6(P) in the ER [84–87] (Figure 6). They proposed that BiP is sequestered to unfolded proteins upon ER stress from pATF6(P), and then pATF6(P) is packaged into COPII vesicles to be transported to the Golgi apparatus. Mori and colleagues reported another mode of regulation of ATF6 activation [88–91]. They found that pATF6(P) forms oligomers through disulfide bonds between cysteine residues in normal growth conditions, and that pATF6(P) oligomers are reduced and become monomers upon ER stress. Thus, they proposed that oligomerization and reduction are involved in the activation process of the ATF6 pathway. Structural analysis of the luminal domain of pATF6(P) would greatly help in understanding the stress-sensing and activation mechanism of the ATF6 pathway.

## The molecular mechanism of pXBP1(U) function

**Figure 7:**
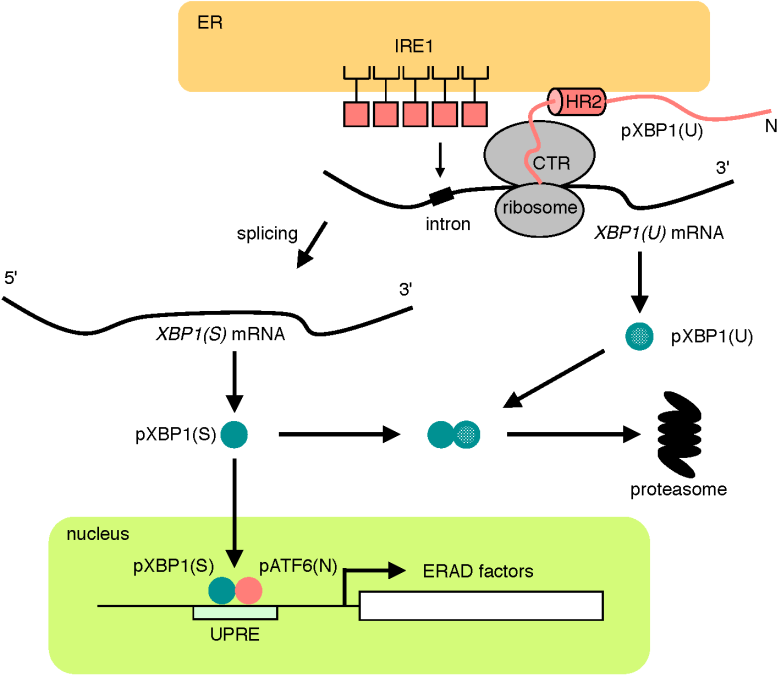
**Three functions of pXBP1(U).** pXBP1(U) translated from *XBP1(U)* mRNA binds to pXBP1(S) and enhances its degradation. The CTR region of pXBP1(U) interacts with the ribosome tunnel and slows translation, while the HR2 region anchors *XBP1(U)* mRNA to the ER membrane, in order to enhance splicing of *XBP1(U)* mRNA by IRE1.

The other issue that would be interesting for structural and computational biologists is the function of pXBP1(U) (Figure 7). In normal growth conditions, *XBP1* mRNA is unspliced and produces pXBP1(U), which contains the DNA-binding domain but not the transcriptional activation domain. Instead, pXBP1(U) has a degradation-enhancing domain, a membrane-association domain and a domain associated with the ribosomal tunnel. During the recovery phase of ER stress, IRE1 is inactivated but pATF6(N) and pXBP1(S) still activate the transcription of ER chaperone genes and the XBP1 gene, which results in an increase in the level of the XBP1(U) transcript and pXBP1(U). pXBP1(U) binds to pATF6(N) and pXBP1(S) and enhances their degradation, leading to the shut-off of the ATF6 and IRE1 pathways [92–94]. In normal growth conditions, the C-terminal region of pXBP1(U) associates with the ribosomal tunnel and reduces the speed of translation [95], while the membrane-association domain of pXBP1(U) binds to the ER membrane [96], resulting in membrane anchoring of *XBP1(U)* mRNA on the ER membrane. This mechanism contributes to the rapid and enhanced splicing of *XBP1(U)* mRNA by IRE1 in response to ER stress. Thus, pXBP1(U) is a multi-functional protein with remarkably interesting functions, but its structure remains to be clarified.

## The mechanism of ER stress-induced apoptosis

The accumulation of unfolded proteins in the ER is very toxic to cells, although the precise mechanism of cytotoxicity caused by unfolded proteins has not been determined [97, 98]. Small oligomers of unfolded proteins are thought to be more toxic than large aggregates, and oxidative stress evoked by unfolded proteins is assumed to be one of the major causes of toxicity.

**Figure 8:**
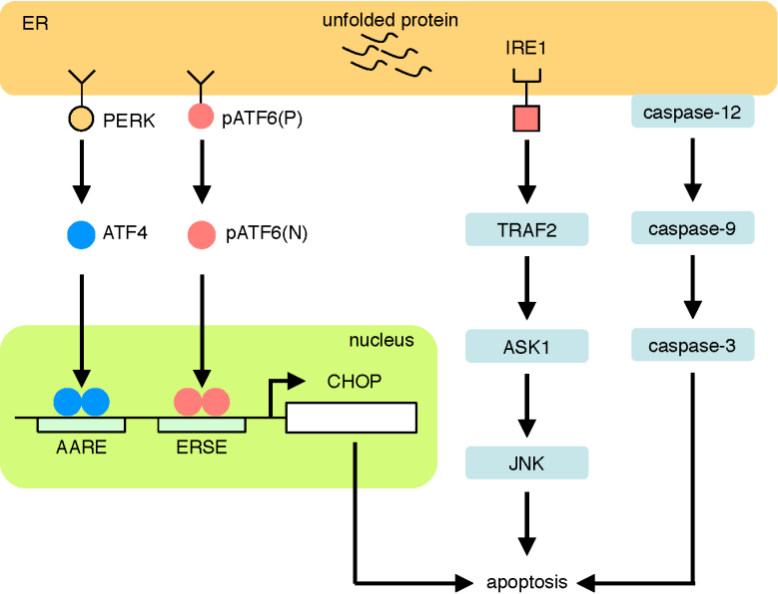
**Major pathways of ER stress-induced apoptosis.** ER stress induces apoptosis through various pathways, including transcriptional induction of CHOP by the PERK and ATF6 pathways, the IRE1-TRAF2 pathway and the caspase-12 pathway.

Of note, the mammalian ER stress response induces apoptotic pathways [3, 99–101] (Figure 8). ATF4 and pATF6(N) bind directly to AARE and ERSE, respectively, in the promoter of the CHOP gene and increase its transcription. CHOP is a well known transcription factor that positively regulates the apoptotic pathway [102–104] and induces transcription of pro-apoptotic genes, including PUMA and BIM [105, 106]. In addition, IRE1 is also involved in the induction of apoptosis. IRE1 activates an apoptotic kinase, JNK, through the signal cascade of the TRAF2-ASK1 pathway [107, 108]. Moreover, murine caspase-12 is also activated upon ER stress and activates the apoptotic pathway; it should be noted, however, that the human caspase-12 gene is a pseudogene [109, 110], and caspase-4 might be involved in ER stress-induced apoptosis in humans [110].

It seems unreasonable that the mammalian ER stress response induces the two opposite responses, namely pro-survival responses and pro-apoptotic responses. However, this is a part of the multi-layered defense system of the mammalian ER stress response, and switching from the adaptive phase to the apoptotic phase seems strictly regulated [111–113]. In acute and mild ER stress, the ER stress response tries to deal with unfolded proteins accumulating in the ER, whereas if cells are damaged by strong and sustained ER stress that they cannot deal with and ER stress still persists and hampers the survival of the organism, the ER stress response activates the apoptotic pathways and disposes of damaged cells from the body. Computational simulation of response pathways to analyze the decision mechanism that determines cell fate (survival or apoptosis) provides a valuable analysis tool, although there have been few such studies to date [114].

Erguler and colleagues reported a mathematical model of the mammalian ER stress response based on the literature, by which they tried to explain how the decision could be made to generate an appropriate response under ER stress conditions of various strengths [115]. The model revealed that the balance between ER stress and the folding capacity of the ER plays a pivotal role in determining cell fate, an adaptive response or apoptosis. There exist three distinct states of behavior, namely, low, intermediate and high activity states. The intermediate state may exhibit oscillations in translation attenuation and apoptotic signals. Though their model integrates the adaptive response mechanisms of the three signaling pathways, their crosstalk, and the associated genetic and post-translational interactions, the signaling cascades of the apoptotic pathway are rather simplified; the model considers only the CHOP cascade. Recently, numerous apoptotic signal cascades have been found to be involved in ER stress-induced apoptosis. For example, calnexin, an ER chaperone specialized for glycoproteins, associates and regulates pro-apoptotic factor Bap31, which is essential for apoptosis [116]. The tyrosine kinase c-Abl translocates from the ER to the mitochondria, resulting in cytochrome c release [117]. Bax inhibitor-1 (BI-1) is an ER protein that suppresses cell death, and BI-1 deficient mice are hypersensitive to apoptosis induced by ER stress [116]. The RING finger E3 ligase RNF186 is localized in the ER and enhances degradation of BNIP1, a member of the Bcl-2 family, in response to ER stress, [118]. miR-106b-25 recognizes the 3’-UTR region of Bim mRNA and suppresses Bim expression. Upon ER stress, the PERK pathway represses miR-106b-25 expression, resulting in increased apoptosis by Bim. Thus, an improved model including these apoptotic factors could significantly advance understanding of the complete decision-making process during the ER stress response.

## Conclusion

The regulatory mechanism of the ER stress response has been analyzed extensively by cell biologists and biochemists, and now the structural and computational approaches are definitely needed for the complete elucidation of the regulatory system as a whole. Recently, homeostatic mechanisms similar to the ER stress response, including the mitochondrial unfolded protein response [119, 120], the lysosomal stress response [121–123], and the Golgi stress response [124], have been reported. The systematic analysis of the homeostatic mechanisms of these organelles will be critical for understanding of the functioning of eukaryotic cells and may provide remarkable contributions to the development of medical science.
